# Antimicrobial effect of herbal and conventional root canal endodontic irrigants against persistent pathogens

**DOI:** 10.6026/973206300191312

**Published:** 2023-12-31

**Authors:** Lalit Kumar Agarwal, Deb Anamika, Rashmi Nair, Shivangi Sharma, Durairaj Preethi, Panikkar Priyanka, Mehta Dhaval Niranjan, Kumar Santosh

**Affiliations:** 1 Department of Conservative and Endodontics, Institute of Dental Sciences, Bareilly, U.P., India; 2 Department of Oral and Maxillofacial Pathology and Microbiology and Forensic Odontology, Bhabha College of Dental Sciences, Bhopal, M.P., India; 3 Department of Conservative and Endodontics, Chhattisgarh Dental College and Research Institute, Rajnandgaon, Chhattisgarh, India; 4 Department of Conservative and Endodontics, D Y Patil School of Dentistry, Navi Mumbai, India; 5 Department of Conservative Dentistry and Endodontics, D. Y Patil School of Dentistry, Navi Mumbai, India; 6 Department of Conservative Dentistry and Endodontics, D. Y Patil School of Dentistry, Navi Mumbai, India

**Keywords:** Antimicrobial efficacy, natural extracts, sodium hypochlorite, persistent root canal pathogens

## Abstract

Evaluation and comparison of natural products like triphala, eucalyptus and carvacol with conventional root canal irrigant such as sodium hypochlorite (NaOCL) and Chlorhexidine against persistent root canal pathogens like *E. faecalis* is of interest. Samples were taken both before irrigation as well as after irrigation. CFU was counted after the plates had been incubated overnight at temperature of 37°C overnight. The herbal products showed antibacterial effectiveness against persistent root canal pathogens like *E. faecalis*. The antibacterial effectiveness was high in NaOCL, chlorhexidine and eucalyptus extract.

## Background:

Antimicrobial endodontic irrigation solutions play a vital role along with biomechanical preparation in elimination of bacteria present in the pulp area to achieve success in endodontic therapy [1-2]. Therefore several antimicrobial endodontic irrigation solutions are applied in conjunction with the biomechanical root canal preparation. At present, the most frequently utilized and widely recognized endodontic irrigant is sodium hypochlorite (NaOCL) having adequate intracanal antibacterial properties against anaerobe *E. faecalis* [3- 4]. There are some disadvantages and short comings of this irrigation solution also. It includes reduction in modulus of elasticity, flexural strength of dentin, elevation in corrosion of instruments for biomechanical preparation of root canals [5-6].

Chlorhexidine [CHX] is a common medication and endodontic irrigant. Its effectiveness stems from the way the negatively charged phosphate groups on the microbial cell walls interact with the positively charged molecule [7- 8], changing the osmotic equilibrium of the cells. As a result, the bacterial cell wall becomes more permeable, facilitating the entry of the CHX molecule. Chlorhexidine gluconate, the most widely used oral formulation, is soluble in water and easily dissociates and releases the positively charged CHX component at physiologic pH [9- 10]. Low molecular weight materials, particularly potassium and phosphorus, will leak out at low concentrations (0.2%). Conversely, CHX is bactericidal at higher concentrations (2%), which precipitate cytoplasmic contents and cause cell death [11-12].

The objective of endodontic therapy is elimination of infectious agents like bacteria and their products from the root canals along with dentinal tubules because these infectious agents are main factors responsible for diseases of endodontic origin [13-14]. Root canal is considered as complicated system due to presence of lateral accessory canals, crossovers of blood vessels, several doors of entry of infections including fins. All these structural features contribute in the accumulation and growth of microorganisms in root canal [15- 16]. It became quite challenging to carry out biomechanical preparation of these root canals as the endodontic irrigants are unable to reach every corner of such canals with complex structure. All these root canals with anatomical complexities may become reservoir of re-infections of root canal [17-18].

The main causative bacteria for such recurrent infections of root canal are Enterobacteria faecalis causing chronic periapical pathology [19-20]. *E. faecalis* is facultative gram positive anaerobic bacteria having physicochemical characteristics like ability to attack dentine, inbuilt resistance power, and ability to form biofilm. These features help *E. faecalis* to survival and grow in adverse conditions [21,22]. Antibacterial properties of natural extracts from leaf of eucalyptus have been reported [18-23, 24]. Antibacterial activities of essential oil residue from leaves of Eucalyptus globules has been reported against *E. coli*, a gram negative bacteria and *S. aureus*, a gram positive bacteria [22,26]. Another herbal medicine is Triphala. It contains *Terminalia bellirica* ("bibhitaki"), *Terminalia chebula* ("halituki") and *Emblica officinalis* ("amulaki"). The citric acid present in fruit assist in removal of smear layer from walls of root canal, thus acting as chelating agent [19,24]. The antibacterial properties against strains of *E. faecalis* by Triphala have been reported elsewhere [25-27]. There is other herbal extract named Carvacrol that raises the non-selective permeability in cell membranes of bacteria. This herbal product is believed to have greater range of action against maximum bacterias [20, 24]. It is found as an important component of organum and essential oilproduced after combination of caustic potash and cymol sulfonic acid [25,28]. There is need for searching other alternatives of root canal irrigation solutions because of some reported drawbacks of conventional irrigation solutions like cytotoxic effects on adjacent tissues, uncomfortable taste, unwanted chemical reactions and increased antibiotic resistance. Hence, evaluation and comparison of natural products like Triphala, eucalyptus and carvacol with conventional root canal irrigant NaOCL and CHX for antibacterial properties against *E. faecalis* is of interest.

## Materials and Methods:

## Preparations of specimens:

## Sample size calculation:

The number of participants was calculated using G*power programme (version 3.0.10) with 0.05 alpha value and 85% power. Power analysis indicated a sample size of at least 179.23 participants. For the study, therefore 180 healthy human premolars of mandible were used. We chose the teeth with single root free of fissures, resorption, grooves as well as canal destruction. The exterior surface of the tooth was cleaned using periodontal curettes, which were then decontaminated in 2.5% NaOCl liquid and stored in a saline solution until needed. All premolar crowns were removed, keeping standardized length of root as15 mm. K-files were used for the BMP up until #20 using tap water for irrigation. The layer that formed the smear was removed using an ultrasound treatment with 17 percent EDTA for ten minutes, then the chemicals were removed using 5.25 percent NaOCl irrigation as well as one hour wash in tap water .To stop microbial spillage, nail polish and resin were applied to the surface and root ends of tooth specimens, respectively. After being placed into glass tubes comprising broth medium with brain heart infusion (BHI), the specimens were sterilised at temperature of 121°C for duration of fifteen minutes. They were then stored in an incubation chamber at temperature of 37°C for duration of 48 hours. *E. faecalis* (ATCC 29212) was procured from the CSIO (Central Scientific Instruments Organization) located in Chandigarh, India, and fully cultivated in BHI 24/7 until the turbidity reached the 0.5 McFarland threshold (1.5 x 108 Colony forming Unit /mL). Glass tubes containing the specimens were opened so that two mL of sterile BHI was substituted with two mL of the bacterial spores, and these tubes were then cultured for duration of twenty-one days at temperature of 37°C. Every two days, the tubes of glass were reactivated to check for bacterial development. Bile esculin examinations, analysis of morphology of colonies in *E. faecalis* broth, and BHI and gram staining were completed. Gram staining was used to confirm the presence of contamination following 21 days. If the teeth were contaminated, they would be removed.

## Triphala:

The Triphala powder used was already prepared and was purchased from IMPCOPS Ltd. located in Chennai. Ten percent DMSO (Dimethyl sulfoxide) marketed by S.D. Fine Chemical Private. Limited, India was used to dissolve Triphala material to create a ten percent triphala endodontic irrigant.

## Natural Extract of eucalyptus leaves:

Eucalyptus plant leaves that were fresh were gathered. The leaves were cleaned with purified water, allowed to air-dry at the ambient temperature, and then grinded. The Soxhlet equipment was then used to retreive the powder with ethanol over the course of twenty four hours. With the use of an evaporator with a rotary blade, the resulting extract was condensed. The extracted substances were once more diluted in DMSO to achieve a 1.25 percent solution.

## Carvacrol:

We used a ready-to-use carvacrol formulation. Carvacrol was mixed in DMSO to modify the needed concentration, and a final concentration of 0.6 percent was obtained.

## Chlorhexidine and Sodium Hypochlorite:

5.25% NaOCl and 0.2% chlorhexidine were used.

## Antimicrobial evaluation:

The research specimens were irrigated with sterilised saline water followed by drying and cleaning with a gauze piece. They were then divided into six categories (n = 30) according to endodontic irrigation solution in following manner:

Category One received 5.25% NaOCl (n = 30)

Category two received 10% Triphala (n = 30)

Category three received 1.25% Eucalyptus extract (n = 30)

Category four received 0.6% Carvacrol (n = 30)

Category five received0.2% chlorhexidine (n=30)

Category Six, saline taken as negative control (n = 30)

Hygienic paper points were placed in each root canal, and then they were moved to a tube containing one mL of BHI & left there for bacteriological testing once the dental samples had been divided into five categories in order to assess and evaluate the antibacterial property of the experimental endodontic root canal irrigants. Then, in accordance with the directions given by the manufacturer, the crown-down approach was utilised to disinfect and shape canals for root canal therapy employing Protaper international rotary files up to F2. Then, utilising a 29-gauge needle, 2 ml of every experimental root canal irrigant was utilised for irrigation of root canal for duration of five minutes before using next instruments. After the application of next instruments of biomechanical preparation, the root canals were then irrigated with four mL of normal saline solution.

## Samples of bacteria:

## Sample from Root Canal:

Following instrumentation, samples were taken from each individual root canal through clean endodontic paper points (Densply). The canal's moisture was absorbed by the paper tips that were subsequently placed in tubes containing 1 mL of broth (BHI). The glass tubing containing the endodontic paper points were placed on a vortex mixer to evenly distribute the microbes on the endodontic paper points. Following that, an inoculation loop was used for streaking. CFU was then seen and counted after the plates had been incubated overnight at temperature of 37°C overnight.

## Specimen from Dentin:

Dentin specimens from walls of root canal were obtained after collecting sample from endodontic root canal. It was carried out through sterile GG drills #3, #4, and #5 (Dentsply, India). Dentine shavings are removed from the wall of root canal by the GG drills #3, #4, and #5 at depths of 200 µm , 400 µm, and 600 µm, respectively. The dentinal scraps from the spiral flutes present on GG drill were collected, which were then streaked on plates after being collected right away in distinct tubes for testing with the aid of a micro-brush. CFU was then measured after the plates spent an entire night in an incubator at temperature of 37°C. The total count of CFU per tooth is calculated by multiplying the total count of colonies of bacteria being detected by the factor of dilution. The whole trial was run under rigorous aseptic conditions in triplicates. To verify the authenticity of the microbial development, the infection's integrity was examined.

## Statistical analysis:

CFU was examined independently. Using SPSS, edition 19.0, descriptive statistics were determined for every category. The statistical significance of antimicrobial effectiveness against *E. faecalis* beforehand and following irrigation was determined using the Wilcoxon signed-rank test. To compare the effectiveness of the six groups' antimicrobial treatments, the Kruskal-Wallis H test was used. P value less than 0.05 was considered statistically significant.

## Results:

The CFU of *E. faecalis* among the specimens of category one (sodium hypochlorite), category two (Triphala), category three (Eucalyptus), category four (carvacrol) and category five (chlorhexidine) and category six (normal saline, negative saline) prior to irrigation was 104.27 ± 19.13, 104.27 ± 19.13, 104.27 ±19.13, 104.27 ±19.13, 104.38 ±19.24 and 104.27±19.13 respectively. On the other hand, while CFU of *E. faecalis* among the specimens of category one (sodium hypochlorite), category two (Triphala), category three (Eucalyptus), category four (carvacrol), category five (chlorhexidine) and category six (normal saline, negative saline) after irrigation was 1.19±3.26, 14.51±15.85, 0.1±0.1, 7.35±17.23, 1.24±0.03 and 59.88±16.78 respectively. There was decrease in CFU post irrigation and the difference was statistically significant (p < 0.001) in each category. During intra group comparisons, there was significant reduction in colonies of *E. feacilis* in each category. When there was inter group comparisons, there was maximum reduction in CFU in specimens of category one (sodium hypochlorite), category five (chlorhexidine) and category three (eucalyptus) and minimum reduction was in category six (negative control). The sequence of reduction in CFU (antimicrobial efficacy) among different categories was

Category three > category one> category five> category four> category 2 > category six

Maximum antimicrobial efficacy was obtained in sodium hypochlorite, chlorhexidine and extracts from eucalyptus. When there was comparison between these three irrigants then there was no statistically significant difference (Table 1). The CFU found in samples of dentin in category one (sodium chlorite), category two (triphala), category three (eucalyptus), category four (carvacrol), category five (chlorhexidine) and category five (negative control) was 0.0852 ± 0.44, 1.333 ± 5.34, 0.068 ± 0.29, 0.392 ± 1.17, 0.0863 ± 0.44 and 0.392 ± 1.17 respectively. The difference in findings was significant statistically (p<0.001). The CFU was in the following order category three < category one < category five < category four < category two < category six (Table 2).

## Discussion:

Due to various documented downsides of standard irrigation solutions, such as cytotoxic effects on surrounding tissues, unpleasant taste, undesired chemical reactions, and increasing antibiotic resistance, there is a need to look for new options to irrigation solutions for root canals. The effects of 0.2% CHX gluconate on infection-ridden root canals were assessed elsewhere [10]. In vitro, Actinomyces (A) israelii-infected root canal walls and dentinal tubules were subjected to a comparison of the disinfectant properties of calcium hydroxide, iodine potassium iodide (IKI), and a CHX solution in study [6- 7]. For 3, 7, and 60 days, the root canals were exposed to calcium hydroxide, IKI, or 2% CHX. The only disinfectant that could completely eradicate *A. israelii* from every sample at every time was CHX [8-9]. A study [18- 20] also reported the antimicrobial effectiveness of Eucalyptus in conjunction with NaOCl, and in their study, they found that NaOCl demonstrated a stronger antibacterial impact against *E. faecalis* as compared to Eucalyptus in contrast to result of present study. This outcome might be explained by the amount of eucalyptus that was employed in the research we conducted, which has the highest antibacterial activity [21-24]. Eucalyptus has demonstrated its antibacterial effect more on gram-positive bacteria such *E. faecalis* in earlier studies [21-23]. Numerous varieties of eucalyptus extracts have been proven through experiments to have antimicrobial effects on *E. faecalis*, Streptococcus mutans, Candida albicans and Lactobacillus [24-25].

After comparing the antibacterial effectiveness of Triphala, 3% of NaOCl, green tea polyphenols on *E. faecalis* biofilms grown on dental substrate [8-12] made the claim that although triphala shown noticeably improved effectiveness against bacteria, it was not as successful as NaOCl. Carvacrol's effectiveness as an additional endodontic irrigation solution against *E. faecalis* was investigated elsewhere [19]. They verified that the endodontic root canals could be adequately cleaned with 0.6% carvacrol. The antibacterial properties against strains of *E. faecalis* by Triphala have been analyzed [15]. There is currently a lot of study being done on the use of natural herbal products as root canal irrigation treatments in endodontic treatment. Natural eucalyptus leaf extracts have been shown to have antibacterial effects in a few studies in the past [12,18]. Essential oil residue from Eucalyptus globules has been shown to have antibacterial properties against the gramme positive bacterium *S. aureus* and the gramme negative bacteria *E. coli* [19-20]. Triphala is a different herbal remedy that has gained attention. Citric acid, which is found in fruit, works as a chelating agent by helping to remove the smear layer from root canal walls [8-15]. At present, the most frequently utilized and widely recognized endodontic irrigant is sodium hypochlorite (NaOCL) having adequate intra-canal antibacterial properties against anaerobe *E. faecalis* [25]. There are some disadvantages and short comings of this irrigation solution also. It includes reduction in modulus of elasticity, flexural strength of dentin, elevation in corrosion of instruments for biomechanical preparation of root canals [26].

Eliminating bacteria and their byproducts from root canals and dentinal tubules is the goal of endodontic therapy because these infectious agents are the primary causes of disorders with endodontic origins. Due to the presence of lateral auxiliary canals, blood vessel crossovers, and multiple entry points for infections, including fins, the root canal system is thought to be complex [10,12]. All of these anatomically complex root canals may develop into a root canal infection reservoir [11]. Enterobacter faecalis, which causes chronic periapical disease, is the principal pathogenic bacteria for such recurrent infections of the root canal. *E. faecalis* is a facultative gram-positive anaerobic bacterium with the capacity to build biofilm and possess physicochemical traits such as the capacity to attack dentine. These characteristics enable *E. faecalis* to survive and develop in challenging circumstances [12-14]. Along with biomechanical preparation, antimicrobial endodontic irrigation solutions are essential for removing germs from the pulp area and ensuring the efficacy of endodontic therapy [15. So, in addition to the biomechanical root canal preparation, a number of antimicrobial endodontic irrigation solutions are used. The prior data were used to calculate the herbal extract's concentration [17-19]. These test substances have demonstrated superior antibacterial activities at specific concentrations in their individual studies, enabling the identification and application of the herbal irrigation solutions with the strongest antimicrobial effect at that concentration against *E. faecalis*, which have been incorporated into the dental model.

## Conclusion:

The herbal products showed antibacterial effectiveness against persistent root canal pathogens like *E. faecalis*. Further, the antibacterial effectiveness was high in sodium hypochlorite, chlorhexidine and Eucalyptus extract compared to other irrigation solutions. However, the effectiveness was statistically similar between sodium hypochlorite, chlorhexidine and Eucalyptus extract.

## Figures and Tables

**Table 1:** Data showing intra group comparisons of CFU in root canals

	**Category one (hypochlorite)**	**Category two (Triphala)**	**Category three (Eucalyptus)**	**Category four (carvacrol)**	**Category five (Chlorhexidine)**	**Category six (negative control)**
Prior to irrigation CFU (Mean±SD)	104.27 ±19.13	104.27 ±19.13	104.27±19.13	104.27 ±19.13	104.38 ±19.24	104.27±19.13
Post irrigation CFU (Mean±SD)	1.19±0.26	14.51±15.85	1.04 ±0.03	7.35±17.23	1.24± 0.03	59.88±16.78
Wilcoxon signed-rank test p-value, significance	W = -6.968	W = -6.954	W = -6.978	W = -6.557	W = -6.971	W = -6.691
	p < 0.001**	p < 0.001**	p < 0.001**	p < 0.001**	p < 0.001**	p < 0.001**

**Table 2:** Data showing CFU in sample from dentin

**Groups**	**Category one (sodium hypochlorite)**	**Category two (Triphala)**	**Category three (Eucalyptus)**	**Category four (carvacrol)**	**Category five (chlorhexidine)**	**Category six (negative control)**
Mean CFU	0.0852*	1.333*	0.068*	0.392*	0.0863*	103.03
SD	0.44	5.34	0.29	1.17		35.08
Kruskal-Wallis H test	H = 1214.4					
p-value,	p < 0.001**					
